# On the Nature and Cure of the Puerperal Fever

**Published:** 1803-07-01

**Authors:** George Mossman

**Affiliations:** Bradford


### *On the* NATURE *and* CURE *of the* PUERPERAL FEVER; *communicated by* Dr. MOSSMAN, *of Bradford.*

IT has been observed that this fever appears on some of the days of the first fortnight after parturition; and that it is more or less fatal according as it takes an early or late date after that period. It exhibits the general characteristics of every proper fever, and is ushered in by similar phenomena. Its approaches are marked by head-ach, lassitude, rigors, flushings of heat, furred tongue, and thirst. Those symptoms are succeeded by nausea, sometimes bilious vomiting, sometimes diarrhœa, pain of the abdominal region, with a soreness extending to the uterus, and frequently to the loins and thighs. The pulse is from 100 to 150, generally feeble and soft; the skin is often dry; the urine is high-coloured, and passed in scanty portions. The most general period of termination is from the fifth to the eleventh or twelfth day. The causes immediately productive of the disease are said to be seven; and every cause has its advocates. If, however, even a very superficial observation shew those causes to be irreconcilable with the acknowledged features of the disease, it ought at least to impeach their validity, and to suggest the probability that they were separately sought up for the purpose of corresponding with a mode of cure previously settled. It is impossible, upon any other supposition, to conceive how causes so different in their operation, and so repugnant to the appearances, both before and after death, could have been adduced to account for the presence of *Puerperal Fever.*

The seven causes are the following:

*An inflammation of the uterus.*
*An inflammation of the intestines and omentum.*
*A stoppage of the lochia.*
*A translation of the milk.*
*A suppuration of the omentum and pus in the abdomen.*
*Infection;* and,
Lastly, *a state of the air favouring the production of this fever.*

If the first of these causes operated in the production of *puerperal fever,* it is proper to expect that *hysteritis* and this disease should correspond most exactly in their symptoms; in the degree of danger, in the mode of cure, and in the appearances after death. For, if a disease be nothing more than an aggregate of certain symptoms, constituting constituting a whole, to which nosologists agree to give a name; and if the symptoms of *hysteritis* and *puerperal fever* be uniformly similar, there needs no other evidence to prove that they are the same disease. This doctrine, however, has never been contended for; nor could it be supported by facts. The symptoms of the two diseases are dissimilar: the degree of danger attached to each is very different; and so also are the remedies employed. I believe that *emetics* are more frequently recommended at the commencement of *puerperal fever* than any other remedy; and there is a very general agreement among authors respecting their beneficial operation. If this statement be correct, it militates strongly against *uterine inflammation* being the cause of *puerperal fever*; for if it were, a little reflection on the action of *emetics*, would serve to demonstrate, that, instead of being subversive of febrile phenomena arising from inflammation, it would very seriously aggravate the evil. No man, surely, would choose to give an emetic in cases of *thoracic* or *abdominal inflammation.*

The *bark of the cinchona too*, has been recommended above every other power, as an agent curative of *puerperal fever.* The evidence of its salutary influence is given by men of high consideration; and the authorities are so respectable in point of talent and experience, that it is difficult to put them down. If the *bark*, therefore, has been administered, and with great and good effect, even at the very commencement of *puerperal fever*, it certainly bears very strongly against the doctrine of *active inflammation* being the cause of the disease. I shall not adduce the evidence of opium, or draw any arguments from the successful administration of this remedy. The question is still agitated, whether its immediate operation be of *sedative* or *stimulant* agency. The experimenters take grounds so opposite, that, at present, it would be unfair to lay hold of either of its supposed powers to affect the present discussion. The enquiry is of high interest, and will, I have no doubt, be soon brought to an issue. There is one point, however, which I can urge with much firmness, and which to my mind affords very conclusive evidence indeed, that the appearances, after death, in cases of *hysteritis* and *puerperal fever*, are widely different. *Effusion, suppuration*, and *gangrene*, embrace, I imagine, the fatal phenomena of *inflammation.* The last of these is found present in fatal cases of *hysteritis*; but I do not know that dissection has ever shewn one well-authenticated cated case of *gangrenous uterus* where the patient perished by the *puerperal fever.* If such a case ever could be brought forward, I apprehend it would prove nothing; for there is an infinity of evidence to establish that *puerperal fever* has proved fatal, where dissection has not been able to shew *inflammation, gangrene,* or *any other morbid appearance of the uterus.*

The same arguments which have been employed to combat the opinion of *puerperal fever* arising from *uterine inflammation,* are also applicable, I think, to the subversion of the second cause which has been assigned; and in addition, it may be observed, that the pains of *intestinal inflammation* are more violent than those which are complained of in *puerperal fever;* and that *bleeding and blistering* have been found effective remedies in the *former,* but not in the *latter* disease.

The third cause, a *stoppage of the lochia,* seems altogether irreconcilable with the facts; for the circumstance of the *lochia* being sometimes absent and sometimes present at the attack, and during the progress of *puerperal fever,* shews their perfect independence of each other. This doctrine wants no additional refutation.

The fourth cause seems to stand on ground equally untenable with that now dismissed. The *milk fever,* (as it is called) appears with features very different from those of the *puerperal fever.* Independent, however, of this dissimilarity, there is one fact which seems decidedly fatal to the doctrine of *puerperal fever* arising from *a translation of the milk,* which is, that this *fever* has been found to attack those women *who have not,* as well as those *who have,* milk in their breasts.

To the fifth cause, it may be objected, that it is impossible for matter to form in the *omentum* so suddenly as to explain the appearances; that were even this supposition probable, the symptoms of *puerperal* do not in any respect resemble those of *hectic fever*; and what puts the matter beyond all doubt, that there has been no appearance of the formation of pus proved by dissection.

The fallacy of the sixth opinion is sufficiently evident from women being seized with *puerperal fever* in situations where contagion could not be supposed to operate or to exist; and from its not seizing upon women who have their abodes with those labouring under the disease. The poison of this contagion would surely not so speedily operate as to be productive of febrile phenomena on the second day; but if it were so pestilential, would it not more more generally seize upon those exposed to its power? It is true, that there is strong evidence of *puerperal fever* being *epidemic* in hospitals and other places; but this proves no more, than that causes similarly circumstanced will very often produce similar effects. The anxiety of women at the period of parturition, the increased irritability of the system, the increased action of the absorbents, the secretion of milk, the too frequent additional degree of heat, applied both in the way of food and cloathing, will all, more or less, share in exciting febrile action, without seeking for any principle of contagion, or accounting for the appearances, on a supposition that certain states of the atmosphere are productive of the disease: and I am fully persuaded, that a cool air in a lying-in chamber, will very generally prevent, and its opposite very frequently induce, the phenomena of *puerperal fever*. It is impossible to account on any other principle for the well-known fact, that *puerperal fever* has been chiefly confined to small apartments and small hospitals; and that since the lying-in chambers have been made more airy and more commodious, and the hospitals larger, the disease has seldom, if ever, been epidemic. It is certainly true, that the most injurious atmosphere to lying-in women, is that created by their attendants.

I am now to speak briefly of the mode of cure, and finish the discussion by considering the *puerperal* as a *proper fever* only, and by advising the varied means which may be applicable to combat the varying symptoms of febrile action

Every author who has seized upon a proximate cause, explanatory of *puerperal fever*, has also laid hold of a correspondent mode of *cure*.

The instruments which have been chiefly employed are the following:

*Bleeding, emetics, laxatives, opium, diaphoretics, camphor, blisters,* the *warm bath,* and *bark*.

*Bleeding*.—If we believe the evidence of authors, this practice has been attended sometimes with beneficial, and sometimes with injurious effects. I am of opinion, however, that the employment of this power, in the commencement of any *fever*, unless where there is local pain, and the existence of inflammation positively ascertained, is always a hazardous, generally an injurious measure. The management of every proper fever, on its accession, requires much caution and skill; and perhaps none so much as the fever under discussion. It was a doctrine among the ancient ancient Jews, "that in the blood was the life;" and were the same doctrine current now, I think with the ingenious John Bell, that "it would be cruel and unnatural to disturb it." I have certainly observed that the commencement of *puerperal fever* is marked by features more strikingly phlogistic than those we meet with in most proper fevers. I am prepared to expect those appearances even in habits of extreme delicacy of fibre; but yet I think the employment of the lancet a most hazardous experiment; for I have observed, that after the third or fourth day, there is a greater prostration of strength, and a greater tendency to *typhus*, than in any other fever. Were I to follow this observation by enunciating an opinion, it would be this, THAT THE LANCET SHOULD NEVER BE EMPLOYED.

If any affection of the chest be connected with *puerperal fever*, or if there be any local pain, I am persuaded that these symptoms may be safely and speedily obviated by the application of *leeches* and *blisters*. In a subject of so much interest, I should wish to be correct; and I really think I have seen as much mischief occasioned by the loss of a few ounces of blood in the commencement of *puerperal fever*, as has followed the loss of the same quantity from the lungs in the last stage of *phthisis*.

*Emetics.*—How emetics operate in the incipient stage of *puerperal* and other *fevers*, I do not determine; but a very enlarged experience has, in my mind, placed their beneficial influence beyond a doubt. The *vinum ipecacuanhæ* is the instrument which I have for many years employed; and in numerous instances I have witnessed its powers to be apparently subversive of the phenomena of fever. In those cases where it has failed to produce this effect, it has, I think, generally lessened the febrile symptoms, and diminished the danger. I conceive *ipecacuanha* to be infinitely more safe, and more solutive of fever than *emetic tartar*. In *puerperal fever*, it is well known that the bowels do very generally partake of the irritability of the system; and the tendency of *emetic tartar* to pass off by the bowels is also well known. The extreme degree of sickness too, which *emetic tartar* frequently produces; and the alarming tumefactions of the abdomen which it sometimes occasions, demand, I think, much caution in its use.

*Laxatives* have been recommended, and they have been reprobated. Their warmest advocates wisely advise the mildest of all. The *oleum ricini* and the *sal catharticus amarus* are of this description, and have been administered for for removing what has been called "the compressing fæces;" for abating the supposed inflammation, and for relieving the distressing head-ach. I am, however, clearly of opinion, that even those weapons, however mild, should be employed by a sparing and by a skilful hand. If they be administered, it should be in the most guarded doses; and their use should never be extended beyond the second or the third day. After those periods (and perhaps at all others in habits of much delicacy) it will be more safe to keep the bowels in a soluble state by means of clysters. It must have occurred to every practitioner to observe, that even small doses of a *cathartic*, in an irritable state of the bowels, have produced such commotion as occasioned much distress, and required potent means to allay.

*Opium* has also been administered in *puerperal fever*; and there is much evidence for and against it. I do not mean now to decide whether it possess *stimulant* or *sedative* powers; I shall only observe, that if it be *stimulant*, this effect is of short duration; for in a few minutes after it has been administered, I know that my patients experience its *composing* or *sedative influence*. I have, perhaps, taken as much *opium* as most men living, and whether I have taken it as an experimenter, or to allay pain (whatever its primary effect may be) I have generally felt its *anoydne* or *soothing* power in the space of a few minutes. In a map of the *materia medica*, I would lay this celestial drug and *digitalis* in the vicinity of each other. In *puerperal fever*, I think, I have administered *opium* with great and good effect. It diminishes the irritability of the system, more especially that of the stomach and bowels. It produces sleep, and excites a gentle perspiration. It lessens the velocity of the pulse; but as the pulse feels stronger, as it is slower, it is not possible to judge of the precise force of the artery without an invention for the purpose.

I have also witnessed the efficacy of *opium* in obviating or in relieving delirium.

*Diaphoretics* have been used, and have been advised by men of high authority. My own experience, however, has taught me to believe, that if they are employed, it should be at the commencement of the fever, and that the selection of the particular instrument employed should be made with great care, and used with great judgment; for if the power employed affect the stomach, or seize on the bowels, it will occasion much mischief, by increasing the irritability of the one, and the debility of the other. Again, if the instrument employed be successful, and if the discharge from from the skin be profuse, and at the same time not solutive of fever, it may impair that vigour which may be wanted at a more advanced period of the disease.

If the cure be attempted by the skin, towards the termination of the fever, it will, by its debilitating operation, infallibly hasten the fatal crisis.

Finally, from what I have observed, I am well persuaded, that if judicious management ever make *diaphoretic* remedies useful, it must be at the very commencement of *puerperal fever*; and that at a more advanced stage, they ought not, under any pretext, to be employed.

*Camphor* has also been administered for the cure of *puerperal fever*; but, as the general *experience* and *experiments* relative to the operation of *camphor*, in this and in other fevers, have not been able to settle its qualities, I think it ought not, by any means, to be admitted to a share of the treatment of a disease so frequently and so speedily fatal.

*Blisters* have had their friends and their enemies; and men of splendid reputations have spoke of them as a beneficial and an injurious application. This want of agreement seems to arise from an anxiety to effect a correspondence between theory and practice. The fact, I am persuaded, is this, that where *blisters* are indicated by the presence of local pain, their employment will seldom disappoint the practitioner; and I know from experience, that where any violent fixed pain has seized upon the abdomen, no power whatever will so speedy, safely, and effectually relieve it as the application of *blisters*; but I also know, that not only in *this*, but in all *proper fevers*, their employment is mischievous, unless indicated by local disease.

The *warm bath* had, and, I believe, still has its partizans; but practitioners, I think, ought to pause before they advise this measure; for much of its operation must hinge on a variety of circumstances; the degree of heat employed; the state of the patient's pulse previous to immersion; the stage of the fever; the habit of body; the duration of the operation; the degree of pain, tension, &c. &c.

It has been observed, that the *warm bath* produces a calm, and disposes to sleep; but is this the effect of exhaustion? Again, is the application of *warm water* primarily *stimulating*, and eventually debilitating in its operation? Further, has it been proved by experiment that the application of *cold water* is solutive of fever? Is it possible that opposite powers should be productive of similar effects?

Upon the presumption of local inflammation in the region of the uterus or abdomen, is heat, in any form, a judicious application? Do we introduce into the room of the patient cool air? Do we remove the bed curtains, and other furniture preventive of a free circulation? Do we remove the heat of the fire? Do we advise cool drinks and a cool regimen? Is the application of hot water either generally or partially, reconcilable with those maxims of cure, and those directions? Finally, have we observed the distressing paroxysms of *gout* and *rheumatism* very constantly increased by hot fomentations, or the application of heat in any form?

I am anxious to dwell on the mischievous effects which I believe to attach to this practice; for none is more common for nurses to employ, or for medical men to enjoin, than hot and moist application to the region of the *uterus*, when even there is any degree of pain or tension in the parts enclosing that organ. It is true in medicine, as well as in other departments of science, that errors are often found to maintain their ground because of their antiquity; for opinions, whether ill or well founded, are subverted with much difficulty, when they have received the sanction of time.

The last remedy which I shall notice is the *bark of the cinchona*. The *bark* has been employed at every period of the fever, and by some, even at its very commencement. There are few men, perhaps, in the profession, who prescribe more *bark* than I do; but I do certainly believe that the *bark* is never to be administered with advantage, seldom with impunity, either in this, or in any other fever, till a remission of symptoms, or a partial subsidence of febrile action has taken place.

It is well understood by every man who has employed the *bark* in the cure of intermittents, that it is never safely nor beneficially employed, till a compleat solution of the febrile phenomena has taken place. When the *cold* and the *hot stages* of *ague* have passed over, then, and certainly not till then, is the *bark* to be administered. In *puerperal fever*, independent of the certainty of its increasing the febrile action, such is the irritable state of the stomach and bowels, that if it be not rejected by the former, it will generally pass off by the latter. I know, by experience, however, that there is a period of this, and every continued fever, when the *bark* of the *cinchona*, judiciously combined and guarded, will exhibit effects strikingly salutary.

I have thus briefly noticed some of the instruments which which are thought to hold the first ranks, as curative agents, in the treatment of *puerperal fever*; and I shall conclude this paper by subjoining a few remarks on the same subject.

By the information which I have collected from authors, from my medical friends, and from my own experience, I am strongly impressed that physicians have been led into error by not treating *puerperal* as a *proper fever* only. They have sought to establish specific causes, productive of specific phenomena, and they have exhibited much anxiety to have *puerperal* considered specifically different from every other *fever*. A close attention, however, to *puerperal fever*, will, I think, shew that it claims all the general features in common with proper fever, with the addition of some circumstance connected with, or consequent of parturition. Observation, practice, and dissection are corroborative of this opinion; and I am convinced that medical efforts would be more successfully directed, were they only exerted to assail symptoms, as is done in the management of proper fever.

I have already observed, that the first approach of *puerperal fever*, (even in constitutions of the greatest delicacy) is marked by a diathesis apparently more phlogistic, and by a greater degree of irritability, than what generally occurs in other fevers; I have also observed that there is a more early change of type, and more early tendency to debility, than commonly occurs in other cases; I have also stated, that the stomach and bowels are seized by a great degree of irritability; that there is generally a soreness of the abdomen, and region of the uterus more or less extended, and often accompanied by an inability to pass the urine. These may be denominated peculiarities; but, with the exception of these, a *puerperal fever* appears to me nothing more than a common continued fever, and ought to be similarly treated. Were it a *symptomatic fever*, as has been contended, the affection of the abdomen would precede the fever, which it certainly does not; and the fever would not be so suddenly fatal.

Upon the whole, I assail *puerperal* with the same confidence that I do any proper *fever*; and tied down by no theory, I combat symptoms as they occur. I employ a *cooling regimen, emetics*, and *laxative clysters*; I give *nitrous* medicines variously combined, with *acids* and *alkalis*, either in a *neutralized* or *effervescing* state. At a more advanced period, I give the *bark* of the *cinchona, always* combined with small doses of *opium*. If ever I employ *blisters*, I do it with a view to remove *local disease*, not to combat

 the the *general operations* of fever. This is briefly the practice I pursue; but as there is still no settled creed respecting the *proximate cause* and the *cure* of *Puerperal Fever*, I hope that some future inquiry will be excited respecting a disease so very fatal, and when so, attended with circumstances peculiarly distressing.

I am, &c.

*Bradford, May* 1, 1803. GEORGE MOSSMAN.

